# Exploiting protein modification systems to boost crop productivity: SUMO proteases in focus

**DOI:** 10.1093/jxb/ery222

**Published:** 2018-06-12

**Authors:** Emma Garrido, Anjil Kumar Srivastava, Ari Sadanandom

**Affiliations:** Department of Biosciences, Durham University, Stockton Road, Durham, UK

**Keywords:** Crop breeding, deSUMOylation, domestication, post-translational modification, SUMO, ULP SUMO proteases

## Abstract

In recent years, post-translational modification (PTM) of proteins has emerged as a key process that integrates plant growth and response to a changing environment. During the processes of domestication and breeding, plants were selected for various yield and adaptational characteristics. The post-translational modifier small ubiquitin-like modifier (SUMO) protein is known to have a role in the regulation of a number of these characteristics. Using bioinformatics, we mined the genomes of cereal and Brassica crops and their non-crop relatives *Arabidopsis thaliana* and *Brachypodium distachyon* for ubiquitin-like protease (ULP) SUMO protease sequences. We discovered that the SUMO system in cereal crops is disproportionately elaborate in comparison with that in *B. distachyon.* We use these data to propose deSUMOylation as a mechanism for specificity in the SUMO system.

## Teaser

Genes encoding deSUMOylating proteases in crop plants are more elaborate than in their non-cultivated relatives, pointing towards a role for deSUMOylation in crop productivity.

## Introduction

The selection pressures applied through centuries-long processes of domestication and breeding of crops have brought about large phenotypical changes. The Brassica genus is a fine example of this process: selection and crossing of ancestral varieties for leaves, stems, flowers, and seeds have led to the development of phenotypically diverse crops such as kale, kohlrabi, cauliflower, and rapeseed, respectively. The cereal family too has seen impactful changes, such as the transition from the low-yielding grass teosinte to the current high-yielding maize varieties (Janick, 2004).

It is a combination of many traits that makes a plant a successful crop. In the case of cereals, all the seed must ripen at approximately the same time and stay on the plant for easy harvesting. Additionally, it must not germinate while still attached to the mother plant or while in storage, but must be reliably viable and germinate quickly and evenly when sown in order to outcompete weeds. As it is the seed endosperm which is destined for consumption, an increased size and/or number of seeds per plant is also desirable (Fuller and Allaby, 2009). Furthermore, levels of defensive toxins must be reduced and the plants must still be able to cope with the biotic and abiotic stresses they encounter in the field.

In recent years, the post-translational modification (PTM) of proteins has emerged as a key process that integrates plant growth and response to a changing environment. It allows for activation or deactivation of stress sensors and downstream transcription factors that control the expression of hundreds of genes. Protein ubiquitination and phosphorylation are the two best understood PTMs controlling stress signalling, but many more exist.

Many key transcriptional regulators including DREB2, ICE1 (controlling cold, heat, salt, and drought stress), NPR1 (regulator of salicylic acid responses), and ABI5 (regulator of abscisic acid responses) have been shown to undergo these PTMs in order to be effective in promoting plant stress adaptation (Miura *et al.*, 2009; Miura and Hasegawa, 2010; Saleh *et al.*, 2015), and PTMs have also been shown to be influential in countering biotic stresses (Casey *et al.*, 2017). PTMs are also relevant to agronomically important alleles. For example, the DELLA genes, which are responsible for dwarfed, high-yielding varieties of the ‘green revolution’ (Peng *et al.*, 1999), undergo multiple PTMs: they have been shown to undergo both ubiquitination (Dill, 2004) and SUMOylation (Conti *et al.*, 2014).

Indeed, SUMOylation is believed to be able to regulate ubiquitination and phosphorylation (Ulrich, 2005; Perry *et al.*, 2008). When ubiquitin is covalently attached to a lysine residue in a substrate protein, this often leads to polyubiquitin chains. Polyubiquitination of a given substrate serves not only as a signal for degradation, but also for retargeting and reprofiling (Hochstrasser, 2009). A key function of the small ubiquitin-like modifier (SUMO) is to act as a vital counterpoise to ubiquitination, adding a layer of control above ubiquitination with respect to substrate availability, stoichiometry, competition for targets, and prevention of ubiquitin-dependent protein degradation (Johnson, 2004). Conversely, SUMOylation can act as a signal for the ubiquitination of proteins by SUMO-targeted ubiquitin ligases (Elrouby *et al.*, 2013). Similarly, recent evidence indicates that phosphorylation can be regulated by SUMOylation of kinases and phosphatases (Crozet *et al.*, 2014), indicating a key point of crosstalk between the different PTMs (Swaney *et al.*, 2013). This places SUMOylation as a likely central regulator of signalling in eukaryotes, and hence is an ideal target for manipulating complex molecular responses such as salt and drought resistance.

SUMO is a conserved protein tag similar to ubiquitin which has been implicated in the plant stress response. Like ubiquitin, SUMO possesses a flexible tail, which allows it to be conjugated to its substrates though an E1–E2–E3 cascade (Saitoh *et al.*, 1997; Hay, 2007). While *Arabidopsis thaliana* encodes two ubiquitin activating enzymes (E1) (Hatfield *et al.*, 1997), up to 45 E2 conjugating enzymes (Chen and Hellmann, 2013), and some 1400 E3 ligases (Hua and Vierstra, 2011), allowing for an extraordinary degree of specificity, the SUMO conjugation cascade encompasses a much smaller group of proteins. Currently only one dimeric E1 SUMO activating enzyme (SAE), one E2 SUMO conjugating enzyme (SCE), and two E3 ligases have been identified in Arabidopsis (Miura and Hasegawa, 2010), suggesting that the specificity of SUMO addition is limited. SUMO chains can then be constructed by the E4 ligases PIAL1 and PIAL2 (Tomanov *et al.*, 2014).

However, SUMOylation is a reversible process. When the stress signal subsides, deSUMOylation takes place. This process is mediated by SUMO proteases. The ubiquitin-like proteases (ULPs) are the most studied family of SUMO proteases. They are cysteine proteases, characterized by a conserved H–D–C catalytic triad (Kurepa *et al.*, 2003). A consensus group of seven ULPs has been identified in Arabidopsis, of which six have been characterized (Murtas *et al.*, 2003; Novatchkova *et al.*, 2004; Conti *et al.*, 2008; Hermkes *et al.*, 2011; Kong *et al.*, 2017; Liu *et al.*, 2017). The observation that substantially more ULPs than E3 ligases are present in Arabidopsis has led to the hypothesis that specificity in the SUMO system may also be conferred by deSUMOylation (Yates *et al.*, 2016). In order to investigate whether this pattern holds true in crops and whether the cultivation level influences the number of ULPs present, we compare the number of putative ULP SUMO proteases in crop plants and their non-crop relatives. Concretely, we compare *A. thaliana* with a number of cultivated Brassicas and *Brachypodium distachyon* with various cereal crops. We hypothesize that some of the selection pressures applied in the domestication and breeding process could provide an insight into the increase in ULP numbers observed in some crops. Additionally, we build on the recent data mined by Augustine *et al.* (2016) on SUMOylating enzymes in cereals to put forward the possibility that the specificity of the SUMO system is at least in part derived from specific deSUMOylation.

## Materials and methods

We used the NCBI-blast (p-blast, DELTA-blast, and PSI-blast) to retrieve protein sequences, using the AtOTS2 catalytic domain as a query for the Brassica crops and the AtOTS2 and BdOTS2 catalytic domains for the cereal crops. As the rice genome is well annotated, we also used the putative function search tool from the Rice Genome annotation project to find putative members of the ULP1 family. Alignements were made using ClustalX and visualized in Jalview. Bootstrap Neighbor–Joining trees were made using ClustalX and visualized using Figtree and MEGA7.

## Results and Discussion

### ULP SUMO proteases in the Brassica family

**Fig. 1.**
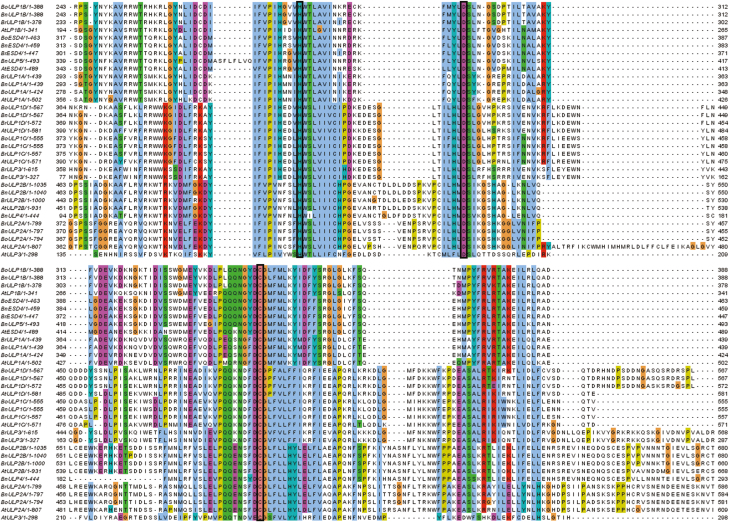
Alignment of Brassica ULP sequences. The H–D–C catalytic triad characteristic of the ULP SUMO proteases is marked in black. The areas surrounding the key amino acid residues show strong conservation across species. Phylogenetically, the Brassica ULPs sort into three branches: ESD4-ULP1A-ULP1B (ESD4 group), ULP1C-ULP1D (OTS group), and ULP2A-ULP2B group (ULP2 group) (Fig. 2). Accession numbers for the proteins used can be found in Supplementary Table S1. Interestingly, both BnULP3 and BrULP3 are part of the OTS group, forming a subclade separate from the ULP1C and ULP1D orthologues, and may themselves be AtULP3 orthologues. Additionally, the two other additional *Brassica napus* ULPs, BnULP4 and BnULP5, are found in the ULP2 and ESD4 groups, respectively.

As the *A. thaliana* proteome is well characterized, we started out by comparing it with the Brassica crops *Brassica rapa* and *Brassica oleracae*, whose cultivars make up most of the vegetable cabbages, and with the oilseed rape *Brassica napus*. The number of sequences retrieved for each species can be found in Table 1. In addition to the seven consensus ULPs, our searches also revealed an eighth ULP candidate, At3g48480. Although originally identified as a ULP candidate (Kurepa *et al.*, 2003), it was not included in the consensus ULP family (Novatchkova *et al.*, 2004) as it is phylogenetically related to a clade of ULP-like genes thought to have emerged from transposon activity (Hoen *et al.*, 2006). However, as At3g48480 encodes a full transcript, has the highest number of associated ESTs seen in this group, and is the most similar to the human ULPs HsSENP6 and HsSENP7 (Lois, 2010)—which are thought to be poly-SUMO deconjugases (Lima and Reverter, 2008)—we included it in our analysis in order to assess whether homologues are present in other Brassica species, naming it AtULP3. The sequences we found to show distinct homology within the catalytic domain (see Fig. 1). Figure 1 reveals that only *B. napus* showed a slight increase in the number of ULPs. As this crop was bred for its seed, we decided to investigate our hypothesis further by focusing on cereal crops, all of which have been bred for their seed.

**Table 1.** ULP SUMO protease sequences retrieved in *Arabidopsis thaliana* and crop Brassicas

Organism	Number of ULP sequences found
***Arabidopsis thaliana***	**8**
*Brassica rapa*	8
*Brassica napus*	10
*Brassica oleracea*	7

As ploidy varies between Brassica species and the sequences were well annotated, sequences identified as isoforms of each ULP were not counted separately. The number of putative ULPs is conserved in *Brassica rapa*, while one sequence less was retrieved in *Brassica oleracea.* Two more sequences were retrieved in *Brassica napus.*

**Fig. 2.**
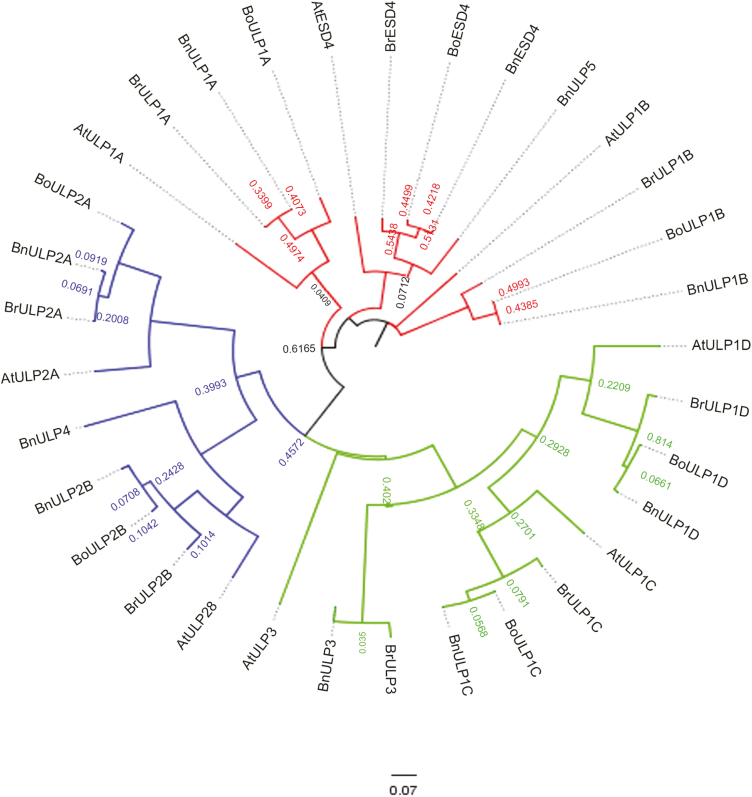
Phylogenetic tree of putative ULP sequences of *Arabidopsis thaliana* and crop Brassicas. The sequences cluster into three groups. The closely conserved ESD4 group incorporates ESD4, ULP1A, and ULP1B homologues and is coloured in red. The OTS group incorporates OTS1 and OTS2 homologues and is coloured in green. The ULP2 group incorporates ULP2a and ULP2b homologues and is coloured in blue.

### ULP SUMO proteases in the cereal family

While the differences in ULP number in the Brassica family are only subtle, those in the cereal family are clearer. Crop plants *Oryza sativa* and *Zea mays* have more than double the number of ULP sequences found in *B. distachyon* (Table 2).

**Table 2.** ULP SUMO protease sequences retrieved in *Brachypodium distachyon* and crop cereals

Organism	Number of sequences retrieved
***Brachypodium distachyon***	**10**
*Oryza sativa*	22
*Zea mays*	21
*Sorghum bicolor*	13
*Hordeum vulgare*	3
*Triticum aestivum*	7

A number of crops encode more putative ULPs than *B. distachyon*, with *Oryza sativa* and *Zea mays* encoding more than double. Due to the underdevelopment of proteome data, very few sequences were recovered from *Hordeum vulgare* and *Triticum aestivum.*

We hypothesize that the reduced increase in ULP sequences found in *Sorghum bicolor* is due to a lack of resolution of the sorghum proteome compared with the highly resolved rice and maize proteomes. This is supported by the fact that we found only three and seven ULP protein sequences, respectively, in barley and wheat, whose proteome data are still underdeveloped. Interestingly, the *Z. mays* genome is thought to stem from a genome duplication event in which an ancestor of *S. bicolor* acted as one of the parent genomes (Swigonova *et al.*, 2004). This duplication may account in part for the lower number of ULP sequences recovered in *S. bicolor* when compared with *Z. mays.* Genes in *O. sativa* do not exhibit a generalized duplication when compared with their *S. bicolor* orthologues (Swigonova *et al.*, 2004). However, the number of ULP sequences recovered from *O. sativa* is roughly equal to that of *Z. mays* rather than *S. bicolor* (Table 2), indicating that multiple factors are likely to be at play.

**Fig. 3.**
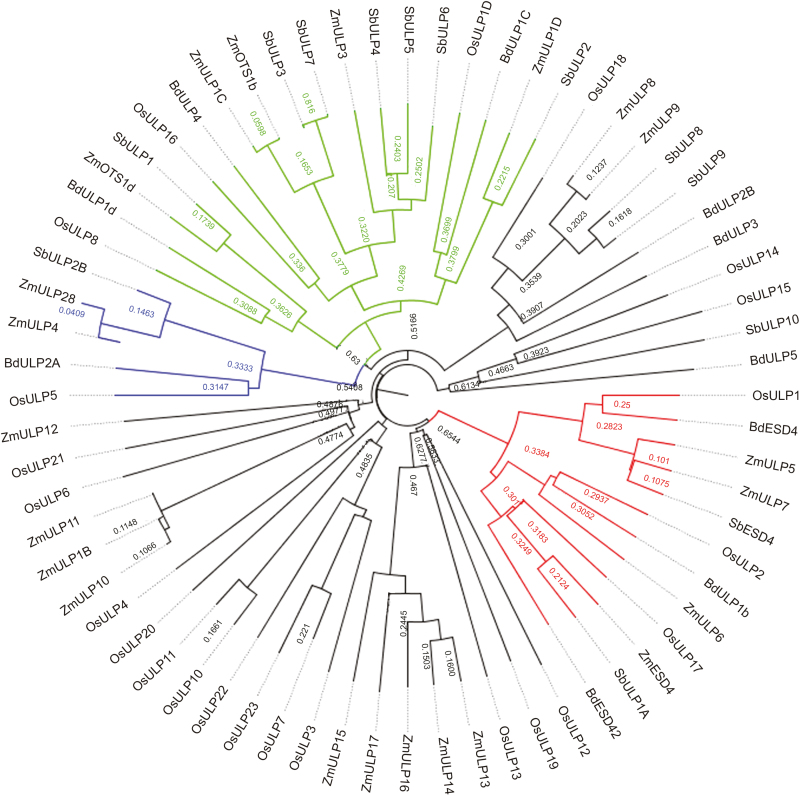
Phylogenetic tree of putative ULP sequences of *Brachypodium distachyon* and crop cereals. The grouping of sequences is less clear in cereals in comparison with Brassicas. This is due in part to the increase in number of sequences and the lack of characterization of crop ULPs. However, the ESD4 group (coloured in red) and the OTS group (coloured in green) remain recognizable. The placement of BdULP2b outside the ULP2 group (coloured in blue) is probably due to the fact that only a partial sequence was recovered. However, new groups have seemingly emerged.

Phylogenetic analysis of the sequences recovered shows partial conservation of the OTS and ESD4 groups (see Fig. 3), but a number of novel groups also emerge, as was previously suggested (Yates *et al.*, 2016). Meanwhile, the ULP2 group is less clearly defined in cereals. Accession numbers for the proteins used can be found in Supplementary Table S1 at *JXB* online.

### The selection pressures applied during domestication and breeding target a number of processes which are known to involve SUMOylation

The centuries-long process of domestication has driven the evolution of low-yielding ancestral plants into the high-yielding crops we know today. This phenotypic transformation is the result of a number of selection pressures exerted in order to make crops easier to grow and harvest, and to improve the yield of the tissue destined for consumption. The main characteristics selected for in cereals have been reviewed both archaeologically and biologically by Fuller and Allaby (2009). They include reducing shattering so as to enable effective harvesting of the seed head and reducing the size and number of seed dispersal appendages to avoid unwanted dispersal by wind or animals. Also important are controlling germination time so as to produce seed that does not germinate in dry storage conditions but germinates promptly and strongly when sown in order to outcompete weeds; increasing seed size and therefore yield, and adapting to changes in environment as the crop spreads to areas by developing ways of coping with new (a)biotic stresses without compromising greatly on yield (Fuller and Allaby, 2009). More fundamentally, the plant must reliably produce viable seed and enough vegetative growth to sustain the development of many and/or large seeds. A number of these aspects have already been linked to (de)SUMOylation, notably fecundity, germination time, seed development, and stress tolerance (Lois *et al.*, 2003; Novatchkova *et al.*, 2004; Saracco *et al.*, 2007; van den Burg *et al.*, 2010; Augustine *et al.*, 2016; Yates *et al.*, 2016).

### Fertility

SUMOylation is involved in various aspects of fecundity, from the ability to form viable embryos to correct flower development and prevention of early flowering.

SUMOylation is known to be essential for embryogenesis: homozygous Arabidopsis mutants for either both the canonical SUMOs SUMO1 and SUMO2, the E1 activating subunit SAE2, or the E2 conjugating enzyme SCE1 are non-viable, aborting in early embryogenesis (Saracco *et al.*, 2007). Additionally, mutants lacking the two known E3 ligases SIZ1 and HPY2 were also non-viable (Ishida *et al.*, 2012). This phenotype coincides with high levels of SUMO1 and/or SUMO2 expression in various parts of developing flowers, seeds, and embryos in wild-type plants as measured by β-glucuronidase (GUS) staining (van den Burg *et al.*, 2010).

Less dramatic but still highly impactful are the single *siz1* and *hpy2* mutants, which were both strongly dwarfed. *hpy2* mutants often do not survive bolting, and a considerable proportion of the seeds they do generate are aborted (Ishida *et al.*, 2012). Not only defective SUMOylation, but also defective deSUMOylation lie at the heart of the dwarfed phenotype. *esd4* mutants are severely dwarfed and early flowering, with a reduced number of flowers and amount of pollen (Reeves, 2002; Murtas *et al.*, 2003; Villajuana-Bonequi *et al.*, 2014). *ots1/ots2* mutants share the first two of these characteristics and exhibit reduced seed numbers in all but ideal conditions (Conti *et al.*, 2008; Campanaro *et al.*, 2016). Fertility is also severely affected in *spf1* mutants, with less than half of seeds developing normally, and in the *spf1/2* double mutant, in which only 15% of seeds complete full normal development (Liu *et al.*, 2017).

One of the causes of the lack of optimal seed development in SUMO mutants could be malformations in floral organ development. For example, *ots1ots2* double mutants exhibit a reduction in stamen elongation which, when rescued by crossing in a DELLA mutation, also restores the otherwise reduced seed formation rate back to wild-type levels (Campanaro *et al.*, 2016). Conversely, some *spf1/2* double mutant flowers exhibit increased style length, causing a physical pollination barrier. Interestingly, this phenotype is only present in approximately one-third of flowers. These double mutants also produced a smaller proportion of viable pollen grains and showed slower pollen tube growth (Liu *et al.*, 2017). Additionally, *siz1* mutants show disrupted guidance of the pollen tube, again reducing the chances of successful fertilization (Ling *et al.*, 2012).

As protein and/or transcript levels of almost all characterized ULP SUMO proteases (the northern blot performed in *esd4* mutants is inconclusive) are known to be elevated in developing flowers (Murtas *et al.*, 2003; Hermkes *et al.*, 2011; Castro *et al.*, 2016; Kong *et al.*, 2017), we suspect the ULP mutants may be harbouring undiscovered floral phenotypes. For example, *esd4* mutants exhibit deformed and irregularly placed siliques (Reeves, 2002) reminiscent of *sum1-1 amiR-SUM2 SUM1/2* knockdown plants (van den Burg *et al.*, 2010), which may be caused by defective flower or seed formation earlier in development.

Complementarily, high levels of SUMO conjugates and SUMOylation elements SUMO1, SAE1, and SCE1 were also observed in flowers (Saracco *et al.*, 2007), emphasizing the critical importance of (de)SUMOylation in floral development.

### Seed size

The end target of cereal breeding of yield is to maximize the amount of seed endosperm produced.. Transcriptional analysis of a range of maize tissues showed endospermal transcriptional up-regulation of the two canonical SUMOs, the maize SAE, a number of SCEs and E3 ligases, and all except two of the eight investigated ULPs (Augustine *et al.*, 2016). Interestingly, the same study found an increase in the transcript levels of SUMO-v, a non-conjugatable SUMO analogue presumably acting through SIM domains in endosperm tissue. The enriched endosperm SUMO system could provide an explanation for the increase in ULP number observed in *B. napus*, a crop bred for its seed, in comparison with other Brassica cultivars.

The difference in the extent of ULP family expansion may be due to the different pathways of endosperm formation and fate observed in monocot grasses and dicot Brassicas (Olsen, 2004). However, very few analyses of the endospermal SUMO system have been published. Furthermore, the data available are limited to transcriptome analysis. Further research into both the transcriptional and (post-)translational level of the endospermal SUMO system in a wider variety of plant species is needed to validate this hypothesis.

### Germination time and stress tolerance

For farmers to be able to differentiate clearly between crop and weed seedlings and for the crop seedling to outcompete their weed competitors, crop seeds must have a narrow germination time window and germinate quickly. Both the *siz1* and the *ots1ots2* double mutant exhibit a late germination phenotype (Castro *et al.*, 2016; Kim *et al.*, 2016) and abscisic acid hypersensitivity (Miura and Hasegawa, 2010), while SUMO overexpression leads to abscisic acid hyposensitivity (Lois *et al.*, 2003).

In the field, crops are grown close together and must be able to withstand the associated abiotic stresses. Moreover, they may have lost defensive toxins in the breeding process, increasing their reliance on other defensive mechanisms to respond to the biotic stresses they encounter in the field. SUMO has long been established as a strong player in the plant stress system.

A variety of abiotic stresses are known to cause the accumulation of SUMO conjugates, including oxidative, salt, osmotic, and temperature stress, which disappear as the plant is given time to recover (Kurepa *et al.*, 2003; Catala *et al.*, 2007; Conti *et al.*, 2008). Meanwhile, *esd4*, *ots1/2*, and *spf1* mutants exhibit higher levels of SUMO conjugation in non-stressed conditions (Conti *et al.*, 2008; Hermkes *et al.*, 2011; Liu *et al.*, 2017), while the ability to recover from stress-induced SUMO conjugate accumulation was shown to be compromised in *spf1* mutants (Kong *et al.*, 2017). Phenotypically, *ots1/2* mutants are more sensitive to salt, osmotic stress, and abscisic acid (Conti *et al.*, 2008; Castro *et al.*, 2016; Srivastava *et al.*, 2017). The salinity tolerance of the *esd4* mutant has not yet been studied but, as it is known to be hypersensitive to abscisic acid (Miura and Hasegawa, 2010), it may also exhibit a salinity phenotype. The mutant phenotypes stand in contrast to the OTS1 overexpressor phenotype, which was shown in both rice and Arabidopsis to be more salt tolerant and to accumulate fewer SUMO conjugates when exposed to stress (Conti *et al.*, 2008; Srivastava *et al.*, 2016).

SUMO also plays a role in the biotic stress system. Both the *ots1/2* and the *siz1* mutant exhibit increased levels of salicylic acid (Jin *et al.*, 2008; Bailey *et al.*, 2016). Salicylic acid is involved in the plant defence against biotrophic pathogens through the mechanism of programmed cell death. While this may confer resistance to *Pseudomonas syringae* to the *ots1/2* (Bailey *et al.*, 2016), it is not necessarily a desirable characteristic. Constitutive hyperaccumulation of salicylic acid pushes the jasmonic acid–salicylic acid antagonism in one direction, leaving the plant unable to mount an effective defence against necrotrophic pathogens.

In addition to SUMO, most of these characteristics share a connection to gibberellin (GA) signalling. The GA–abscisic acid equilibrium mediates germination, the endosperm being the key seed layer in the perception of this equilibrium (Nambara *et al.*, 2010). The DELLA proteins, which are degraded in response to GA, are known SUMOylation targets and play a role in flower development (Campanaro *et al.*, 2016), growth repression in abiotic stress situations (Conti *et al.*, 2008), shade avoidance, and in the equilibrium between growth and defence against biotic stresses mediated by GA and jasmonic acid (Pieterse *et al.*, 2014). All this information leads us to hypothesize that the key to uncovering the importance of SUMOylation in crops may lie in the identification of SUMO targets in GA-related proteins.

### A number of cereal-specific SUMO system components provide a basis for a more complex SUMO system in cereals

Characterization of the SUMO system in cereals has led to the discovery of a number of components not present in dicots. First, a peptide similar to di-SUMO was identified to be expressed at low levels in the maize female gametophyte (Srilunchang *et al.*, 2010) and later in the male gametophyte (Augustine *et al.*, 2016).

Secondly, the cereal family was found to contain a new subclass of SCE proteins. Active site modelling revealed an increased proportion of negatively charged amino acids around the active site, suggesting that they may exhibit altered specificity (Augustine *et al.*, 2016). However, the number of neither class I nor class II SCEs is consistently more elevated in cereal crops than in *B. distachyon* (Table 3).

**Table 3.** Number of crop SCE proteins found by Augustine *et al.* (2016)

Organism	Class I	Class II
*Arabidopsis thaliana*	1	0
*Brachypodium distachyon*	2	1
*Oryza sativa*	2	1
*Zea mays*	4	3
*Sorghum bicolor*	2	3

Cereals have a higher number of (putative) SCE proteins than *Arabidopsis thaliana*. Specifically, class II SCEs are only found in cereals. However, crop cereals do not consistently encode more SCEs than *Brachypodium distachyon*

### DeSUMOylation as a mechanism for specificity in the SUMO system

Even when the increased numbers of SCE enzymes in cereals are taken into account, the ULP SUMO proteases outnumber the SUMOylating enzymes (see Tables 1–3). This raises an interesting question with regard to specificity within the SUMO system: specificity may be imparted by deSUMOylation rather than by SUMOylation, especially as the ULP SUMO proteases are unlikely to be the only class of SUMO proteases in plants. Two other classes of SUMO proteases have previously been identified in mammals (Hickey *et al.*, 2012). As the PTM process is conserved in eukaryotes, they may also be present in plants. Further investigation of these classes in plants could turn the ubiquitin-based model of specificity in post-translational peptide tags upside down.

### Conclusion

We used bioinformatics to investigate the number of ULP SUMO proteases in crops and their non-crop relatives. Within the cereal family, we found a substantial increase in rice and maize when compared with Brachypodium. This increase could be due to the selection pressures applied during the domestication and breeding processes heavily involving processes in which SUMO is known to be involved. However, most of our knowledge about the SUMO system comes from Arabidopsis and requires verification in crops. In cereals, the endospermal SUMO system in particular must be further characterized.

We further hypothesize that deSUMOylation by the ULP SUMO proteases and other as yet uncharacterized SUMO proteases could provide a mechanism for specificity within the SUMO system alongside the multiple SUMO tags and E3 ligases. As the ULPs are involved in both yield and stress resistance, they could provide a possible target for the generation of high-yielding stress-resistant crops. However, further research is needed to characterize the specific targets of each of the ULPs.

## Supplementary data

Supplementary data are available at *JXB* online.

Table S1. Accession codes of the crop ULP SUMO proteases used in Fig. 3

Supplementary Table S1
